# Association of PPE Availability, Training, and Practices with COVID-19 Sero-Prevalence in Nurses and Paramedics in Tertiary Care Hospitals of Peshawar, Pakistan

**DOI:** 10.1017/dmp.2020.438

**Published:** 2020-11-05

**Authors:** Junaid Ahmad, Saeed Anwar, Abdul Latif, Najib U. Haq, Muhammad Sharif, Ahmed A. Nauman

**Affiliations:** 1 Prime Institute of Public Health, Peshawar Medical College, Peshawar, K.P.K., Pakistan; 2 Peshawar Medical College, Peshawar, K.P.K., Pakistan

**Keywords:** COVID-19, PPE, training, practices, sero-prevalence, Pakistan

## Abstract

**Objective:**

Nurses and paramedics by being the frontline workers of the health-care profession need to be equipped with the relevant knowledge, skills, and protective gears against different forms of infection, including coronavirus disease 2019 (COVID-19). Although the governments and concerned stakeholders have provided personal protective equipment (PPE), training and information to protect the health-care professionals; however, until now the scientific literature has virtually not reported the impact of PPE availability, training, and practices on the COVID-19 sero-prevalence among the nurses and paramedics. This study aimed to assess the impact of PPE availability, training, and practices on COVID-19 sero-prevalence among nurses and paramedics in teaching hospitals of Peshawar, Pakistan.

**Methods:**

A cross-sectional survey was conducted with a total of 133 nurses and paramedics as subjects of the study.

**Results:**

A univariate analysis was done for 4 variables. The findings indicate that the health-care professionals (nurses and paramedics) who have received PPE on time at the start of COVID-19 emergence have fewer chances of contracting the COVID-19 infection (odds ratio = 0.96); while the odds for PPE supplies was 0.73, and the odds of hand hygiene training was 0.95.

**Conclusions:**

The study concluded that the availability of the PPE, COVID-19–related training, and compliance with World Health Organization recommended practices against COVID-19 were instrumental in protection against the infection and its spread.

Pandemics and epidemics are not something new to humanity. Several devastating epidemics, pandemics happened and were recorded in the past.^1^ Just in the past couple of decades, since the beginning of 21st century, more than 6 major epidemics and pandemics have struck humanity in different parts of the world including the Ebola virus epidemic, Zika virus epidemic, Dengue virus epidemic, H1N1 virus epidemics, Middles East respiratory syndrome (MERS) epidemics, and the current pandemic of coronavirus disease 2019 (COVID-19).^2^ Mostly, the major pandemics in the past were caused by influenza viruses. Among all the recorded pandemics, the most iconic pandemic is the “Spanish flu” that hit the world in 1918 and resulted in an estimated 17-100 million deaths.^3-5^ The current COVID-19 pandemic is the worst in recent history, which until now has impacted more than 186 countries across the globe and shut down almost every business both in the developed and developing country.^6^

COVID-19 has emerged not only as a public health issue, but also a geopolitical one.^7^ Governments, oppositions, and organizations are using the COVID-19 associated aspects, especially mortality and morbidity rates to safeguard their public and political images.^8^ One such issue is the availability of personal protective equipment (PPE) for health-care professionals.^9^ The issue has been highlighted a lot all over the electronic and social media that governments failed to provide the frontline health-care professionals with enough PPE, resulting in health-care professionals being more vulnerable and at higher risk of contracting COVID-19 compared with the general public.^7-9^ This is reported both in developing and developed countries. Although much is being discussed both at official and nonofficial platforms about the lack of PPE provision, however, the evidence-based figure has rarely been reported about the COVID-19 prevalence in health-care professionals.

Just as all over the world, the pandemic of COVID-19 also severely affected Pakistan. Pakistan, a South-Asian country, is a neighboring country to the 2 of the worst affected countries by the COVID-19, that is, The Peoples Republic of China (the epicenter of COVID-19) and the Islamic Republic of Iran (one of the worst-hit COVID-19 countries). Although the COVID-19 originated and initially spread from the Peoples Republic of China, Pakistan recorded its first confirmed COVID-19 case as an imported case from the Islamic Republic of Iran on February 26, 2020.^8^ By August 30, Pakistan recorded a total of 295,896 confirmed COVID-19–positive cases and 6294 COVID-19–induced deaths throughout the country. The health-care professionals in Pakistan are the hard-hit segments of society by the COVID-19, as, by June 30, a total of 5367 health-care professionals contracted the coronavirus infection. Among all the health-care professionals who became infected with COVID-19, doctors make the significant chunk, as 61% (3275 out of 5367) of the total were doctors, followed by the paramedical staff at 27% (1453 of 5367), and nurses at 27% (639 of 5367).^10^ The province of Khyber Pakhtunkhwa (K.P.K.) in general as well as in terms of health-care professionals is the worst affected province of the country in terms of COVID-19 infections. The province makes up only 11% of the total country population, but 33.66% (1807 of 5367) of health-care professionals infected with COVID-19 were from K.P.K.^11^

Since the newly emerged pandemic of the COVID-19, much has been discussed about its place of origin, etiology, clinical features, mortality and morbidity rates, etc.^12,13^ Also, much has been emphasized that health-care professionals are the frontline warriors and are at high risk of contracting the COVID-19.^14^ However, studies that reported the prevalence of COVID-19 in health-care professionals rarely exist, and even the limited number of available studies are restricted to retrospective and symptomatic cases only.^15-17^ It is worth mentioning that, until today, not a single study has reported the impact of PPE availability, training, and practices on COVID-19 sero-prevalence in health-care workers, including nurses and paramedics.

## Methods

This study is a part of a larger study (*N* = 1012)^18^ by the investigators that aim at studying seroprevalence for COVID-19 among the various health-care providers working in a set of 3 selected public and private sector teaching hospitals of Peshawar, Pakistan. The larger study (not published yet) has found a comparatively very high prevalence among nurses and paramedics. This study using both descriptive and analytical techniques aimed to determine if the availability of PPE, COVID-19 infection prevention and control training, and compliance with the World Health Organization (WHO) recommended practices was in any way related with seroprevalence among the nurses and paramedics. A cross-sectional study was conducted with 38% participation rate (aggregate of the nurses and paramedics; *N* = 350). The study subjects were telephonically interviewed by means of a pretested structured questionnaire, designed explicitly for the purpose, after informed verbal consent.

Data entry and analysis were done with the help of IBM SPSS Statistics V22.0. The socio-demographic variables included the profession with a breakdown by age and gender. Significance testing (chi-squared test) was done, or odds ratios were calculated for categorical variables and other variables of interest to identify the protective and risk factors among the subjects.

### Study Area

The study was conducted in the metropolitan city Peshawar, a capital city of the Khyber Pukhtunkhwah (K.P.K.). This is one of the most marginalized and underprivileged provinces in the country, which has been very hard hit by conflicts, wars, and natural disaster for the past 40 y. The city is hosting public (governments), private (for-profit), and charity (not-for-profit) hospitals. The total population of the city is approximately 1.97 million, and the total documented beds in the hospitals are approximately 8000.

### Inclusion Criteria

Nurses and paramedics who were full-time and fully-paid employees of the participating hospitals and consented voluntarily to participate in the study were included in the study.

### Institutional Review Board Approval

The Institutional Ethical Review Board of Peshawar Medical College gives exemption from full review for this study based on no intervention involved in the study.

## Results

### Demographic information

A sub-sample of 350 nurses and paramedics was drawn from the earlier phase study sample (N_1_ = 1012) for telephonic interview. All nurses and paramedics who were tested for COVID-19 antibodies were contacted through telephonic calls. A structured text message indicating the purpose of the study and voluntary participation, along with ensuring participants confidentiality and anonymity, was delivered to all the participants a day before the interview. All the eligible participants (N_2_ = 350) were contacted through phone calls; however, with a 38% response rate, only 133 participants responded to the questionnaire. Of them, 36% (*n* = 48) participants were nurses and 64% (*n* = 85) paramedics. Among the nurses, diploma holders were 75% (*n* = 36), and only 25% (*n* = 12) were graduates in nursing

Paramedics were also predominantly diploma holders 85% (*n* = 72), while only 15% (*n* = 13) were degree holders. The majority of our study participants were male, 89% (*n* = 118). Compared with 33% of the females, 41% of males have reactive antibodies against COVID-19. A significant difference in seropositivity was observed among the age group 15-24 y (24%, *n* = 63) and 25-55 y (52%, *n* = 67). Sero-positivity in nurses and paramedics were 48% (*n* = 23) and 41% (*n* = 35), respectively (Table 1).

**Table 1.** Descriptive statistics of the demographic profile

	Health-Care Worker	Sero +Ve *n* (%)	Sero −Ve *n* (%)	Total
Profession	Nurses	23 (48%)	25 (52%)	48
	Degree holder (n = 12)	6 (50%)	6 (50%)	12
	Diploma holder (n = 36)	17 (47%)	19 (53%)	36
	Paramedics (n = 85)	35 (41%)	50 (58.8%)	85
	Degree holder (n = 13)	5 (38%)	8 (62%)	13
	Diploma holder (n = 72)	30 (42%)	42 (58%)	72
Gender	Male	48 (41%)	70 (59.3%)	118
	Female	5 (33%)	10 (67%)	15
Age	15-24	15 (24%)	21 (33%)	63
	25-50	35 (52%)	41 (61%)	67
	65-Above	0	4 (100%)	4

### Practices of the Participants

Descriptive statistics were calculated for practices of the study participants regarding COVID-19 infection prevention comprising 9 areas of possible risk factors. For each, multiple variable responses (always, most of the time, never, sometimes, and occasionally) were recorded to achieve a real situation of the practices by the respondents.

The data analysis revealed that 55% (*n* = 73) of the study participants went to crowded places during an active pandemic, and 45% (*n* = 59) used public transportation during this time. Always wear a mask when leaving home was reported by 78% (*n* = 102), while careful removal of the protective gear was reported by 61% (*n* = 81) of the participants.

Furthermore, 79% (*n* = 105) of the participants stated that, during coughing and sneezing, they cover their mouth and faces. Washing hands with soap and water were reported by 77% (*n* = 102) while washing hands after returning home with soap and water were recorded by 87% (*n* = 116) of the participants. Only 52% of the study participants always complied with the most critical preventive practice against COVID-19; social distancing. Surprisingly, although 51% (*n* = 57) of the participants have reactive antibodies against COVID-19, only 9.8% (*n* = 13) of the participants self-isolated at home during the pandemic (Table 2).

**Table 2.** Descriptive statistics of practices as reported by the study participants

Have you gone to any crowded place during the COVID-19 pandemic?	Always	1.5% (n = 2)
	Most of the time	3% (n = 4)
	Never	45.1% (n = 60)
	Occasionally	42.1% (n = 56)
	Sometimes	8.3% (n = 11)
	Total	100% (n = 133)
Do you wear a mask when leaving home?	Always	76.7% (n = 102)
	Most of the time	5.3% (n = 7)
	Never	0.8% (n = 1)
	Occasionally	9.8% (n = 13)
	Sometimes	6.8% (n = 9)
	Total	100% (n = 133)
Did you remove protective equipment carefully as a routine?	Always	60.9 % (n = 81)
	Most of the time	4.5% (n = 6)
	Never	0.8% (n = 1)
	Occasionally	24.1% (n = 32)
	Sometimes	9.8% (n = 13)
	Total	100% (n = 133)
Do you cover mouth when coughing and sneezing during this pandemic?	Always	78.9% (n = 105)
	Most of the time	8.3% (n = 11)
	Never	2.3% (n = 3)
	Occasionally	7.5% (n = 10)
	Sometimes	3% (n = 4)
	Total	100% (n = 133)
Have you used public transport during the COVID-19 pandemic?	Always	13.5% (n = 18)
	Most of the time	3% (n = 4)
	Never	55.6% (n = 74)
	Occasionally	18.8% (n = 25)
	Sometimes	9% (n = 12)
	Total	100% (n = 133)
Do you wash hands with soap and water?	Always	76.7% (n = 102)
	Most of the time	8.3% (n = 11)
	Never	0.8% (n = 1)
	Occasionally	11.3% (n = 15)
	Sometimes	3% (n = 4)
	Total	100% (n = 133)
Do you wash hands after returning home?	Always	87.2% (n = 116)
	Most of the time	4.5% (n = 6)
	Occasionally	5.3% (n = 7)
	Sometimes	3% (n = 4)
	Total	100% (n = 133)
Do you comply with the recommended social distancing for COVID-19?	Always	52.6% (n = 70)
	Most of the time	7.5% (n = 10)
	Never	4.5% (n = 6)
	Occasionally	19.5% (n = 26)
	Sometimes	15% (n = 20)
	Total	100% (n = 133)
Did you take care of self-isolation or quarantine at home also during the pandemic?	Always	9.8% (n = 13)
	Most of the time	7.5% (n = 10)
	Never	58.6% (n = 78)
	Occasionally	19.5% (n = 26)
	Sometimes	4.5% (n = 6)
	Total	100% (n = 133)

### Relationship of Early Availability of PPE and COVID-19–Related Training with Sero-prevalence in Health-Care Workers

Univariate analyses were done for 4 variables as potential risk factors for contracting COVID-19 infection. The absence of adequate and timely supplies of PPE and lack of infection control training, including COVID-19–related training, were considered to be risk factors along with unsafe practices in acquiring COVID-19 infection in health-care workers. Odds ratios (ORs; q_x_) were calculated to determine any association among the mentioned variables, and whether sero-prevalence exists. Findings suggest that 3 variable (PPE timely and adequate availability, COVID-19–related training, and hand hygiene training) had a positive association with seronegativity. Health-care workers who have received PPE on time at the start of COVID-19 emergence had less chance of acquiring COVID-19 infection (OR = 0.96). There is a mild protective association among timely PPE availability and seronegativity; however, other confounding factors, such as environmental factors, cannot be ruled out. Similarly, health-care workers who had not faced any interruption of PPE supply had comparatively lesser reactive COVID-19 antibodies (OR = 0.733). Adequate and timely availability of PPE has mild protective effects on COVID-19 infection. COVID-19–related training, including PPE use (donning, doffing), social distancing, isolation, quarantine, lockdown, had a protective association with COVID-19 infection (OR = 0.98). There was no difference in the odds for the training and sero-prevalence between the diploma holders and graduate health professionals. Furthermore, health-care workers who had received training on hand hygiene had less chances of getting COVID-19 infection (OR = 0.95) (Table 3).

**Table 3.** Association of early availability of PPEs and COVID-19–related training with sero-prevalence in health-care workers

		Antibody Test Result				
		−Ve	+Ve	Total	Risk Estimates/OR	95% Confidence Interval
Did you get the PPEs early during the pandemic?	No	24	19	43	0.966	(0.464-2.009)
	Yes	51	39	90		
		75	58	133		
Were PPEs provided in adequate amount?	No	10	10	20	0.733	(0.104-2.04)
	Yes	65	48	113		
		75	58	133		
Did you obtain COVID-19 related training, such as PPE use (donning, doffing), social distancing, isolation, quarantine, lock-down, etc.?	No	32	25	57	0.982	(0.492-1.96)
	Yes	43	33	76		
		75	58	133		
Did you obtain hygienic hand washing during the past 3 years?	No	29	23	52	0.959	(0.475-1.93)
	Yes	46	35	81		
		75	58	133		

## Discussion

This study is unique, because it assessed the impact of PPE availability, training, and practices on COVID-19 sero-prevalence in nurses and paramedics in teaching hospitals of Peshawar-Pakistan. However, a lot of research has been conducted and much can be found in the published literature about the efficacy of mask use in the prevention of COVID-19, the usefulness of handwashing in avoiding the transmission of COVID-19, and efficacy of information dissemination in minimizing the spread of COVID-19. However, limited information is available when it comes to establishing as to what is their impact on the sero-prevalence among health-care workers, including nurses and paramedics.

The inadequate supply of PPE is/was an issue not only in developing countries but also developed countries also faced such hurdles. Like, the study reported that the high prevalence rate of COVID-19 in health-care professionals is partly attributed to the lack of adequate supply of PPE to health-care workers.^18^ The findings of our study are also fully aligned with the finding reported by Ranney et al.^19^ Whereas, COVID-19 is surrounded by many uncertainties in terms of its place of origin, etiology, clinical features, mortality rates, morbidity rates, risk prevention strategies, etc., this study is of paramount importance, as it will initiate a debate among the policy-makers and concerned stakeholders to take into account the protection of nurses and paramedics with the existing recommended preventive measures as well as the launch of additional interventions to make sure that a package comprising the adequate supply of PPE, tailored infection prevention and control training, and access to updated personal protection protocols by the WHO is made available to them.

## Conclusions

Based on the findings of this study, it can be concluded that COVID-19 sero-prevalence in nurses and paramedics is directly determined by PPE availability, training, and practices in tertiary care hospitals. The health-care workers in general, and specifically nurses and paramedics, will always be at high risk for contracting the COVID-19 if they are not provided with an adequate supply of PPE, up to date infection control training, and proper infection prevention practices. There is no doubt that, in the current age of unprecedented pandemic of COVID-19, governments and concerned stakeholders are under pressure to provide adequate supply of PPE to protect health-care worker against COVID-19; however, there is no short-term and shortcut solution to tackle the spread of COVID-19, other than to make sure PPE availability, regularly conduct infection control training, and enforce WHO recommended practices.
